# Targeting high Aurora kinases expression as an innovative therapy for hepatocellular carcinoma

**DOI:** 10.18632/oncotarget.15853

**Published:** 2017-03-02

**Authors:** Fuchen Liu, Guangyong Wang, Xiaoqiang Wang, Zhihui Che, Wei Dong, Xinggang Guo, Zhenguang Wang, Ping Chen, Daisen Hou, Qi Zhang, Wenli Zhang, Yida Pan, Dongqin Yang, Hui Liu

**Affiliations:** ^1^ The Third Department of Hepatic Surgery, Eastern Hepatobiliary Surgery Hospital, Second Military Medical University, Shanghai 200438, China; ^2^ Department of Digestive Diseases of Huashan Hospital, Fudan University, Shanghai 200032, China; ^3^ Department of Gastroenterology, 411 Hospital of PLA, Shanghai 200081, China; ^4^ Department of Neurosurgery, Xinhua Hospital, Shanghai Jiaotong University School of Medicine, Shanghai 200092, China

**Keywords:** Aurora A/B kinases, SNS-314, HCC, YAP, P21

## Abstract

The Aurora kinases A and B control tumorigenesis by inhibiting apoptosis and promoting proliferation and metastasis, however, it remains unknown whether Aurora A and B overexpressed concomitantly and its clinical significance in hepatocellular carcinoma (HCC). Here, we obsearved Aurora A and B tended to overexpress parallelly on protein level (*r* = 0.8679, *P* < 0.0001) and their co-overexpression (Aurora A^H^B^H^), associated with the worst prognosis, was an independent predictor for the survival. Importantly, with the lower IC50 and stronger anti-tumor effect than selective inhibitors, SNS-314, the pan-inhibitor of Aurora kinases, which induced YAP (Yes-associated protein) reduction and resulted in P21 accumulation, significantly promoted the polyploidy (> 4N) formation and apoptosis in HCC. High YAP expression (YAP^H^) was associated with Aurora A^H^B^H^, and appeared to be an independent predictor for survival, but P21 not. Moreover, silencing YAP also induced P21 accumulation, and knockdown P21, which enhanced YAP accumulation and weakened the SNS-314-induced YAP reduction, impaired SNS-314-induced apoptosis. Therefore, P21 enhanced the apoptotic effect of SNS-314 in HCC. Taken together, our findings indicated Aurora kinases/YAP/P21 was an oncogenic signaling axis in HCC, and revealed targeting Aurora A^H^B^H^ induced apoptosis by YAP suppression. Our results also provided a solid evidence for SNS-314 as a potential targeted therapy, and a proof-of-concept evidence for a possible combined therapy of SNS-314 plus Hippo pathway inhibitors on HCC.

## INTRODUCTION

Hepatocellular carcinoma (HCC) can be seen as a prototypical therapy-resistant tumor, causing the third leading mortality of malignancies [1, 2]. As the first line treatment for advanced HCC, benefits of sorafenib was limited due to the severe adverse events [3, 4]. Previous study shows the multitarget kinase inhibitors for Aurora kinases and Vascular Endothelial Growth Factor Receptor 2 (VEGFR2) imporve antitumor activity over sorafenib in HCC [5].

The Aurora kinases, belonging to serine-threonine kinase family, including Aurora A, B and C, control chromosome assembly during mitosis [6, 7]. Aberrant expression of Aurora kinase A and B has been reported in a variety of solid tumors including colon [8], pancreas [9] and breast [10, 11], which indicates Aurora kinases are involved in tumorigenesis and tumor development [12, 13]. Aurora C has attracted much less interest due to its expression limited to testis [6]. Currently, there are several Aurora kinase inhibitors undergoing phase I and II clinical studies for solid cancers [14, 15]. These findings have led to an increased interest in these kinases as their molecule inhibitors have been developed as latent anticarcinogen.

Aurora kinase A directly phosphorylated Lats2 to interact with Hippo pathway [16, 17]. However, the significance of the relationship between Aurora kinases and Hippo pathway has not been illuminated in HCC.

In this study, we determined the relationship of Aurora A and B expression, and analyzed the significance of their concomitant high-expression on prognosis in HCC. We also provided the evidence that the combined treatment, pan-Aurora kinases inhibitor plus Hippo pathway inhibitor, could be a more effective therapy for HCC.

## RESULTS

### Both Aurora A and B protein were overexpressed in parallel in HCC and their high expression predicted a poorer prognosis

**Figure 1:**
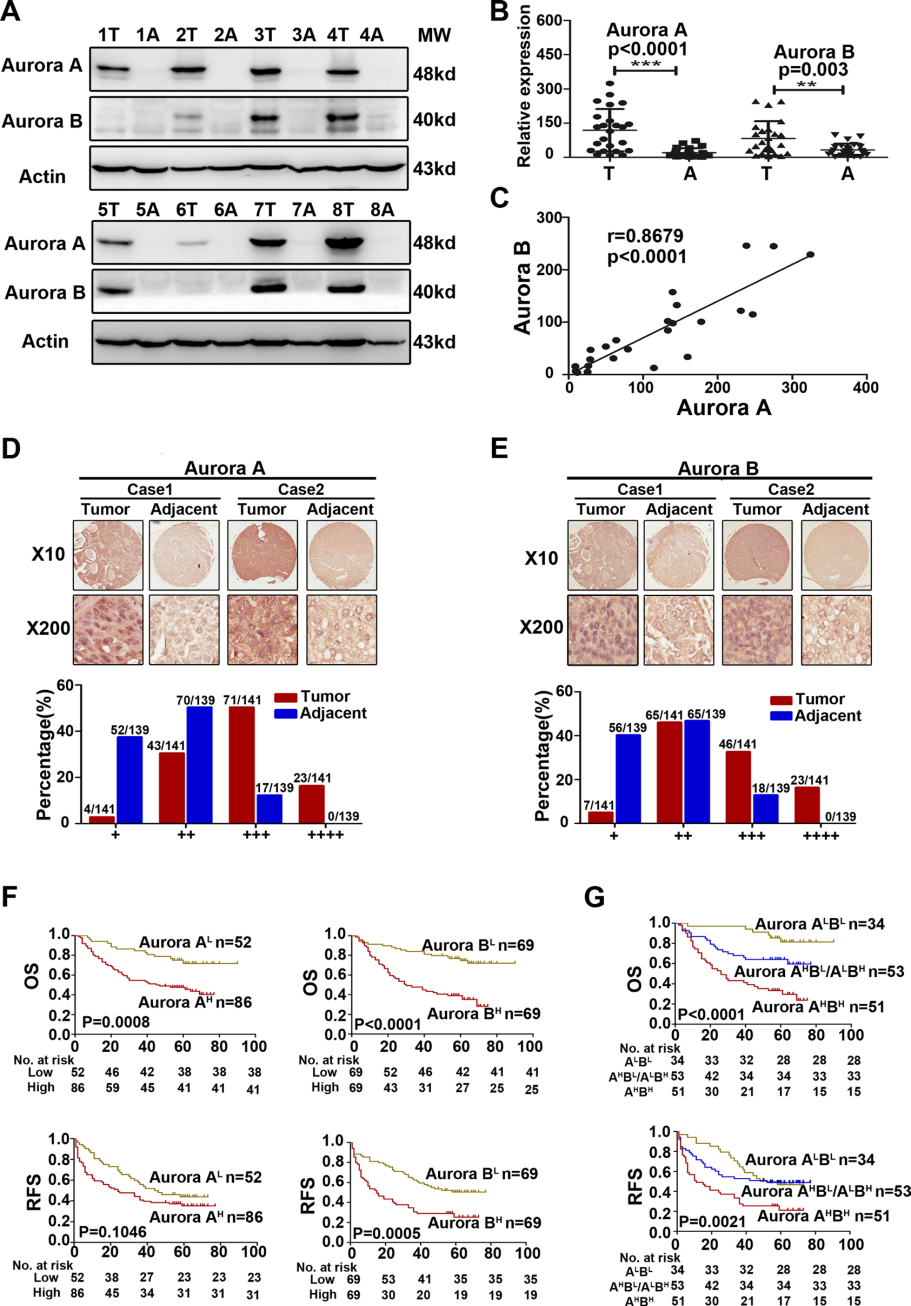
Aurora A and B were overexpressed in parallel and predicted a poorer prognosis in HCC (**A**) WB analysis to determine the expression of Aurora A and B in HCCs and adjacent liver tissues and representative results of 8 out of 24 pairs of tissues were shown. (**B**) Quantification of Aurora A and B expression in HCCs and adjacent liver tissues (*n* = 24). (**C**) A positive linear correlation relationship existed between Aurora A and B at the protein level. (**D–E**) IHC staining of human HCC tissue array using Aurora A and B-specific antibody, respectively, as described in Material and Methods. And the classification of samples according to the intensity of staining of Aurora A and B expression, respectively. (**F**) Survival correlation analysis of Aurora A and B expression status in HCC, respectively (*n* = 138). (**G**) Survival correlation analysis among the groups of Aurora A^High^B^High^ (Aurora A^H^B^H^), Aurora A^High^B^Low^/A^Low^B^High^ (Aurora A^H^B^L^/A^L^B^H^) and Aurora A^Low^B^Low^ (Aurora A^L^B^L^), with different Aurora expression status (*n* = 138). OS, overall surivival rate; RFS, relapsed free survival rate; **p < 0.01, ***p < 0.001.

To assess Aurora kinases expression in HCC tissues, we examined the expression of Aurora A and B protein in 24 cases of HCC matched with adjacent tissues by Western blot, and found that the expression of Aurora A and B were both significantly higher than those of corresponding adjacent tissues (Aurora A *P* = 0.0001, Aurora B *P* = 0.003) (Figure 1A–1B). We also found a positive linear correlation between Aurora A and B at protein level (*r* = 0.8679, *P* < 0.0001, Figure 1C).

The data from Oncomine Database showed that the mRNA level of Aurora A and B in HCC cancer tissues were all higher significantly than that of the normal or adjacent tissues (Supplementary Figure 1). We further analyzed Aurora A and B expression in 141 cases of HCC, matched with 139 adjacent tissues, by immunohistochemical (IHC) staining. The results showed that Aurora A and B were expressed primarily in nucleus, and Aurora A highly expressed in the tumor tissues compared with adjacent tissues. Based on the staining intensity of IHC, the samples were classified into four groups from weakest group 1(+) to strongest group 4 (++++). If Aurora A expression was weak, falling into groups 1 and 2, otherwise, if its signal was strong, falling into groups 3 and 4. In groups 1 and 2, the majority of adjacent normal tissues (52/139 and 70/139) had weak expression of Aurora A compared to tumor tissues (4/141 and 43/141); however, in groups 3 and 4, the majority of liver cancer tissues (71/141 and 23/141) had strong expression of Aurora A compared to adjacent normal tissues (17/139 and 0/139) (Figure 1D). Similar results were observed for Aurora B expression in the same cohort (Figure 1E). Importantly, the high expression of both kinases exhibited the positive linear correlation in tumor tissues (*r* = 0.2380, *P* = 0.0264), which further confirmed the result of WB analysis (Figure 1C).

We analylzed the relationship between Aurora kinases expression and patients’ survival, and found that patients with high Aurora A or B expression (Aurora A^H^, Aurora B^H^) had significantly shorter overall surivival rate (OS) or relapsed free survival rate (RFS) than that with low expression (Aurora A^L^, Aurora B^L^)( Figure 1F). We classified the patients into three groups based on the Aurora A and B expression: both Aurora A and B high expression group (Aurora A^H^B^H^), Aurora A or B high expression group (Aurora A^H^B^L^/A^L^B^H^) and both Aurora A and B low expression group (Aurora A^L^B^L^). It was showed that 37% (51/138) patients with Aurora A^H^B^H^ expression was in our cohort (Table 1), and Kaplan–Meier analysis indicated that the group patients had much shorter OS and RFS than that of the other groups (Figure 1G). The association of their expression with survival was further confirmed by multivariate analysis, suggesting that Aurora A^H^B^H^ expression was an independent predictor for OS and RFS (Table 2).

**Table 1:** Clinical features of 138 HCC patients and Aurora A and B expression

Feature	Aurora A and B expression number *n* = 138				*p* value
		(A^L^B^L^) *n* = 34(25%)	(A^H^B^L^/ A^L^B^H^) *n* = 53(38%)	(A^H^B^H^) *n* = 51(37%)	
**YAP**					0.004
	Low	26 (76%)	25 (47%)	21 (41%)	
	High	8 (24%)	28 (53%)	30 (59%)	
**Age, Years**					0.246
	< 50	10 (29%)	25 (47%)	22 (43%)	
	≥ 50	24 (71%)	28 (53%)	29 (57%)	
**Child-Pugh**					0.329
	A	33 (97%)	47 (89%)	45 (88%)	
	B	1 (3%)	6 (11%)	6 (12%)	
**AFP, ng/ml**					0.19
	< 20	12 (35%)	13 (25%)	21 (41%)	
	≥ 20	22 (65%)	40 (75%)	30 (59%)	
**HBsAg**					0.051
	(−)	8 (24%)	6 (11%)	3 (6%)	
	(+)	26 (76%)	47 (89%)	48 (94%)	
**Major tumor size (diameter, cm)**					0.994
	< 5	15 (44%)	24 (45%)	23 (45%)	
	≥ 5	19 (56%)	29 (55%)	28 (55%)	
**Number of nodules**					0.929
	< 2	26 (76%)	39 (74%)	39 (76%)	
	≥ 2	8 (24%)	14 (26%)	12 (24%)	
**satellites nodules**					0.467
	NO	6 (18%)	13 (25%)	15 (29%)	
	YES	28 (82%)	40 (75%)	36 (71%)	
**MVI**					0.792
	NO	13 (38%)	19 (36%)	16 (31%)	
	YES	21 (62%)	34 (64%)	35 (69%)	
**HBeAg**					0.532
	(−)	30 (88%)	42 (79%)	43 (84%)	
	(+)	4 (12%)	11 (21%)	8 (16%)	

*P* < 0.05 was considered statistically significant. Pearson's chi-square test was used.

Abbreviations: YAP, Yes-associated protein; AFP, Alpha Fetal Protein; HBsAg, hepatitis B surface antigen; MVI, Microvascular invasion; HBeAg, hepatitis B e antigen.

**Table 2:** Uni- and multivariate analyses of factors associated with survival and recurrence in 138 HCCs

Factors	OS				RFS			
	Univariate, *P*	Multivariate			Univariate, *P*	Multivariate		
		HR	95% CI	*P*		HR	95% CI	*P*
**Age, Years (< 50 vs. ≥ 50)**	0.99			NA	0.675			NA
**Child (B vs. A)**	0.286			NA	0.047	1.951	1.063–3.580	0.031
**AFP (< 20 vs. ≥ 20)**	0.215			NA	0.425			NA
**HBsAg (positive vs. negative)**	0.712			NA	0.466			NA
**Major tumor size (diameter, cm)**	0.009	1.074	1.016–1.134	0.011	0.038	1.066	1.011–1.124	0.018
**Number of nodules (< 2 vs. ≥ 2)**	0.172			NA	0.452			NA
**Satellites nodules (YES vs. NO)**	0.487			NA	0.646			NA
**MVI (YES vs. NO)**	0.562			NA	0.547			NA
**HBeAg (positive vs. negative)**	0.057			NA	0.078			NA
**Aurora A/B**								
**A^H^B^L^/A^L^B^H^ VS. A^L^B^L^**	0.33			NS	0.829			NS
**A^H^B^H^ VS. A^L^B^L^**	< 0.001	2.442	1.533–3.888	< 0.001	0.011	1.753	1.108–2.776	0.017
**YAP (High vs. Low)**	0.001	1.533	1.059–2.219	0.024	0.025	1.474	1.029–2.112	0.035
**P21 (High vs. Low)**	0.191			NA	0.228			NA

Cox's proportional hazards regression model.

Abbreviations: OS, overall survival; RFS, relapsed-free survival; NA, not adopted; NS, not significant; YAP, Yes-associated protein; AFP, Alpha Fetal Protein; HBsAg, hepatitis B surface antigen; MVI, Microvascular invasion; HBeAg, hepatitis B e antigen; 95% CI, 95% confidence interval; HR, hazard ratio.

Moreover, we observed the association of Aurora A and B expression with clinicopathological features in HCC. Aurora A and B expression was positively correlated with HBsAg level, as 48 out of 51 patients with Aurora A^H^B^H^ was HBsAg (+), but only 26 out of 34 patients with AuroraA^L^B^L^ or 47 out of 53 patients with Aurora A^H^B^L^/A^L^B^H^ (Table 1). Taking all apsects of the results into account, our results suggested the oncogenic effect of high Aurora A and Aurora B expression (Aurora A^H^B^H^) in HCC.

### The novel pan-Aurora kinases inhibitor SNS-314 suppressed the proliferation of liver cancer cells

**Figure 2:**
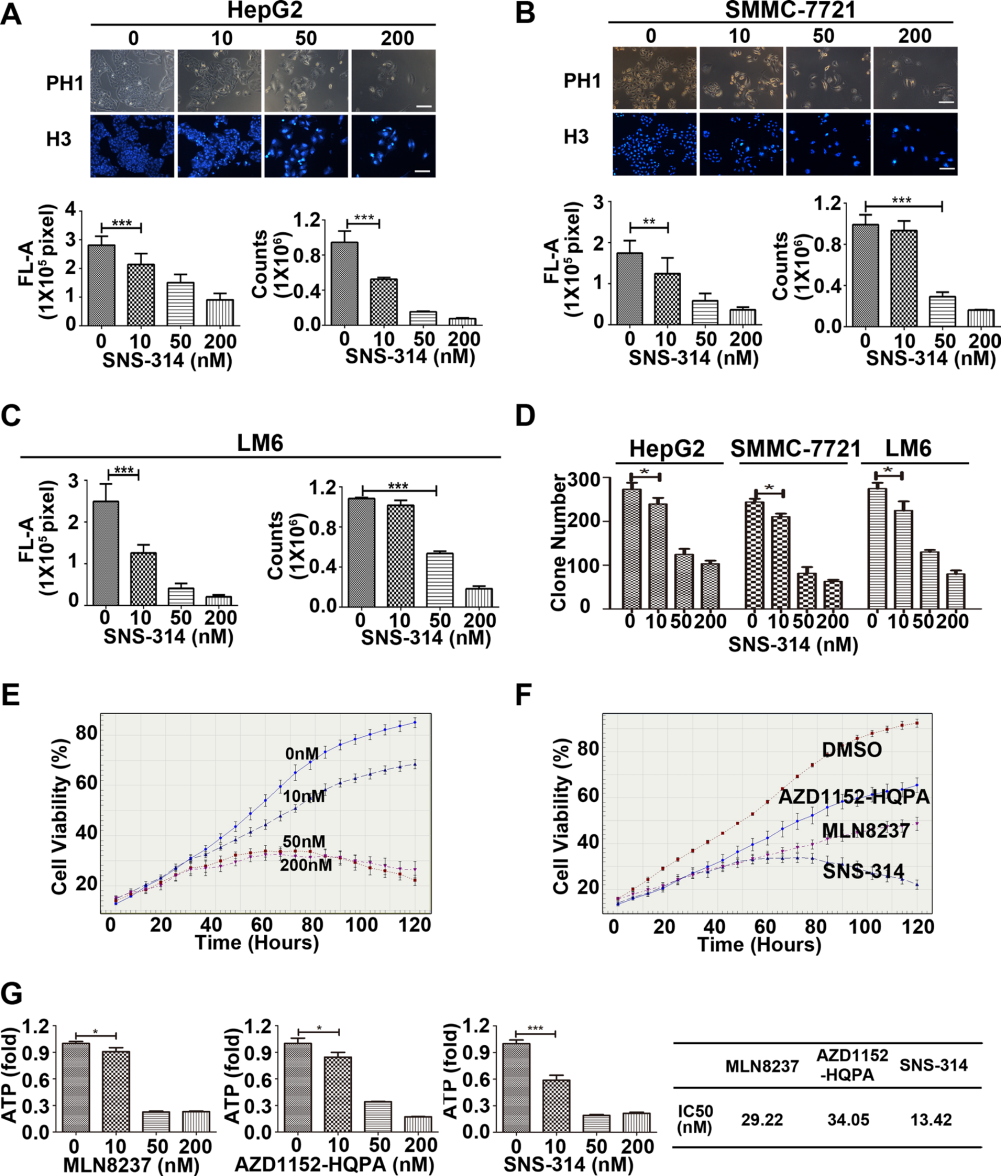
The growth-suppressive effect of inhibitors of Aurora kinases on HCC cells (**A–C**) SNS-314 inhibited the proliferation of HepG2 (A), SMMC-7721 (B) and LM6 (C) cells under the indicated concentrations for 96h, then stained with Hoechst 33342 and analysed according to the Material and Methods, and representative images were shown in HepG2 and SMMC-7721; or trypsinized and counted at each time point (*n* = 3). (**D**) SNS-314 affected the colony-formation ability of liver cancer cells. HepG2, SMMC-7721 and LM6 cells were treated under the indicated SNS-314 concentrations for 10 days and then subjected to colony-formation assay (*n* = 3). (**E**) HepG2 were treated with SNS-314 and monitored in real time by the live-cell imaging system IncuCyte™. Representative growth curves of cells treated with SNS-314 at the indicated concentration were shown. Each curve was performed at least four times, and each time point was determined in triplicate. (**F**) HepG2 were treated with SNS-314, MLN8237 and AZD1152-HQPA (50 nM), and monitored in real time by the live-cell imaging system IncuCyte™. Representative growth curves of treated-cells with the indicated time were shown. Each curve was performed at least four times, and each time point was determined in triplicate. (**G**) The IC50s of Aurora inhibitors, MLN8237, AZD1152-HQPA, and SNS-314, in HepG2 treated for 96 h measured by ATPlite assay. H3, Hoechst33342, FL-A, Fluorescence-Area; Mean ± SD; *p < 0.05, **p < 0.01, ***p < 0.001.

To further verify the oncogenic effect of Aurora A^H^B^H^ in liver cancer cells, we observed the effect of SNS-314 (a novel pan-Aurora kinases inhibitor [18]) on cell proliferation of 3 liver cancer cell lines (SMMC-7721, HepG2, LM6) by Hoechst 33342 staining, cell counting and colony-formation assay. The results showed that SNS-314 treatment for 96 h significantly reduced the Hoechst staining, cell counts and clone numbers in a dose-dependent manner (Figure 2A–2D). The IncuCyte™ real-time imaging system was used to monitor the growth curve of HepG2 cells, which also showed that the inhibition effect of SNS-314 was significant in dose- and time-dependent manners (Figure 2E). With IncuCyte™ real-time imaging system and ATP cell viability assay, we compared the effect of SNS-314 on HCC proliferation with the treatment of specific inhibitors of Aurora kianse A (MLN8237) or B (AZD1152-HQPA [19]); and the results showed that with lower IC50 SNS-314 inhibited the cell proliferation more effectively than the kinase specific inhibitors (Figure 2F–2G). These results implicated that the pan-inhibition of both Aurora A and B kinases has the stronger anti-proliferation effect than that of single kinases in liver cancer cells, further surported the strong oncogenic effect of Aurora A^H^B^H^ expression in liver cancer.

### SNS-314 promoted the polyploidy formation and apoptosis of HCC cells

**Figure 3:**
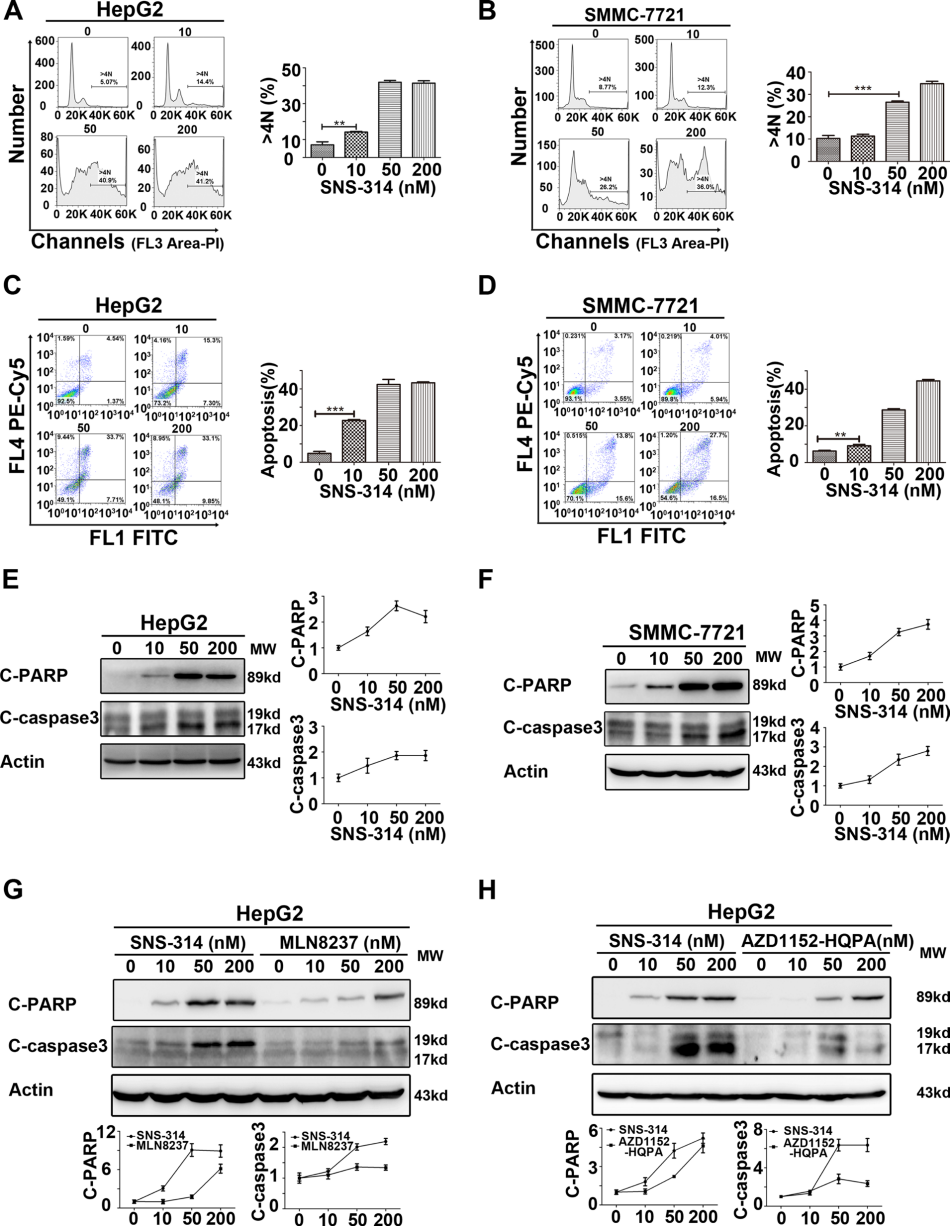
Polyploidy and apoptosis of HCC cells were induced by the inhibitors of Aurora kinases (**A–B**) Polyploidy (DNA>4N) of HepG2(A) and SMMC-7721(B) cells was induced by SNS-314 in a dose-dependent manner, which was showed by PI staining to determine the cell cycle profile. (**C–D**) Apoptosis of HepG2(C) and SMMC-7721(D) cells was induced by SNS-314 in a dose-dependent manner, which was showed by PI and Annexin V staining. (**E–F**) Expression of apoptotic proteins following SNS-314 treatment in HepG2(E) and SMMC-7721(F) cells suggested by WB analysis and quantified by the Image J software. (**G–H**) The expression of apoptotic proteins induced by SNS-314 was much higher than MLN8237 and AZD1152-HQPA in HepG2 cells which was suggested by WB analysis and quantified by the Image J software. FL, Fluorescence; C-PARP, cleaved-PARP; C-caspase3, cleaved- caspase3; Mean ± SD; **p < 0.01, ***p < 0.001.

To understand the mechanism underlying SNS-314-induced cell proliferation arrest, we analyzed the cell cycle profile and apoptotic rate in HCC cells treated with SNS-314. PI staining showed that the percentage of the polyploidy (DNA>4N) formation was significantly increased in SNS-314-treated HepG2 (Figure 3A) and SMMC-7721 cells (Figure 3B) in a dose-dependent manner; the percentage of apoptotic cells (Annexin V (+)) was also significantly increased in a dose-dependent manner (Figure 3C–3D). Moreover, the activation of c-PARP (cleaved poly-ADP-ribose polymerase) and c-caspase3 (cleaved-caspase3) was further confirmed by WB (Figure 3E–3F). Importantly, the accumulation of c-PARP and c-caspase3 in HCC cells treated with SNS-314 was much higher than that of the cells treated with MLN8237 or AZD1152-HQPA, respectively (Figure 3G–3H). Therefore, these results indicated SNS-314-induced cell proliferation arrest of HCC through polyploidy formation and apoptosis, which can also explain why SNS-314 has stronger effect than that of the single kinase inhibitors.

### Downregulation of Aurora kinases suppressed the YAP activity and resulted in the P21 accumulation

**Figure 4:**
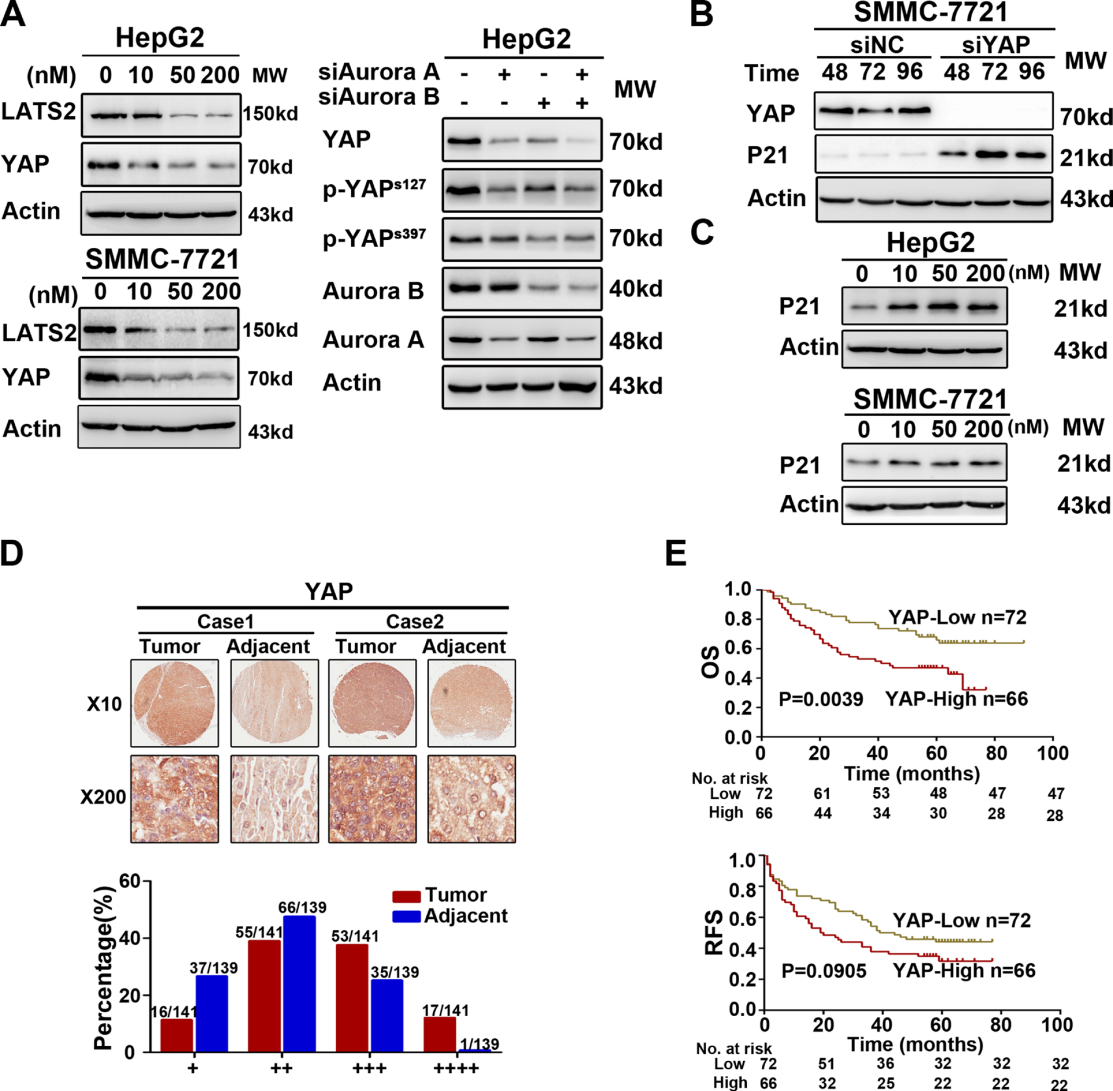
Interference of Aurora kinases contorlled the Hippo pathway and resulted in the accumulation of P21 (**A**) The key proteins of Hippo pathway was suppressed due to the knockdown or inhibition of Aurora kinases in HepG2. (**B**) YAP knockdown induced the accumulation of P21 via WB analysis in SMMC-7721. (**C**) The accumulation of P21 was induced significantly by SNS-314 treatment with indicated concentrations for 96h in HepG2 and SMMC-7721 cells. (**D–E**) IHC staining of human HCC tissue array using YAP-specific antibody, as described in Material and Methods. And the classification of samples according to the intensity of staining of YAP expression (*n* = 138). OS, overall surivival rate; RFS, relapsed free survival rate.

Previous studies demonstrated that Aurora A and Hippo pathway could interact each other [16, 17], and our results showed that both MLN8237 and AZD1152-HQPA reduced LATS2 and YAP protein level (Supplementary Figure 2). As the major downstream effector of the Hippo pathway, YAP functions as an oncoprotein [20]. To further understand the molecular mechanisms underlying SNS-314-induced apoptosis, we observed YAP was significantly reduced in HCC cells upon Aurora kinases knockdown or SNS-314 treatment (Figure 4A). Due to the essential of YAP for P53-P21 pathway suppression [21, 22], YAP silencing resulted in the P21 accumulation (Figure 4B). Furthermore, SNS-314 treatment also induced the P21 accumulation in HepG2 and SMMC-7721 cells (Figure 4C).

We next assessed YAP expression in the above 141 cases of HCC. According to IHC staining, YAP expression level was classified into four groups (group 1–4). The YAP expression, which were high in most tumor tissues, falled into group 3 (+++) or 4 (++++), whereas YAP expression, which were low in majority of tumor tissues, were classified into group 1 (+) or 2 (++) (Figure 4D). We also analyzed the expressed relationship between YAP and Aurora kinases, and found that the number of the patients with high YAP expression (YAP^H^) was significantly less in the patients with Aurora A^L^B^L^ (24%) than that with Aurora A^H^B^H^ (59%) and AuroraA^H^B^L^/A^L^B^H^ (53%), respectively (*P* = 0.004, Table 1). Kaplan–Meier analysis indicated that YAP^H^ expression was associated with briefer OS (YAP *P* = 0.0039) compared to that of YAP^L^ expression (Figure 4E). Multivariate analysis determined YAP^H^ was an independent predictor for poor OS (*P* = 0.024) and RFS (*P* = 0.035) (Table 2). These results suggested that inhibition of Aurora kinases suppressed YAP activity, which may have additional benefits for HCC management.

### Knockdown of P21 impaired the SNS-314-induced apoptotic effect in the liver cancer cells

**Figure 5:**
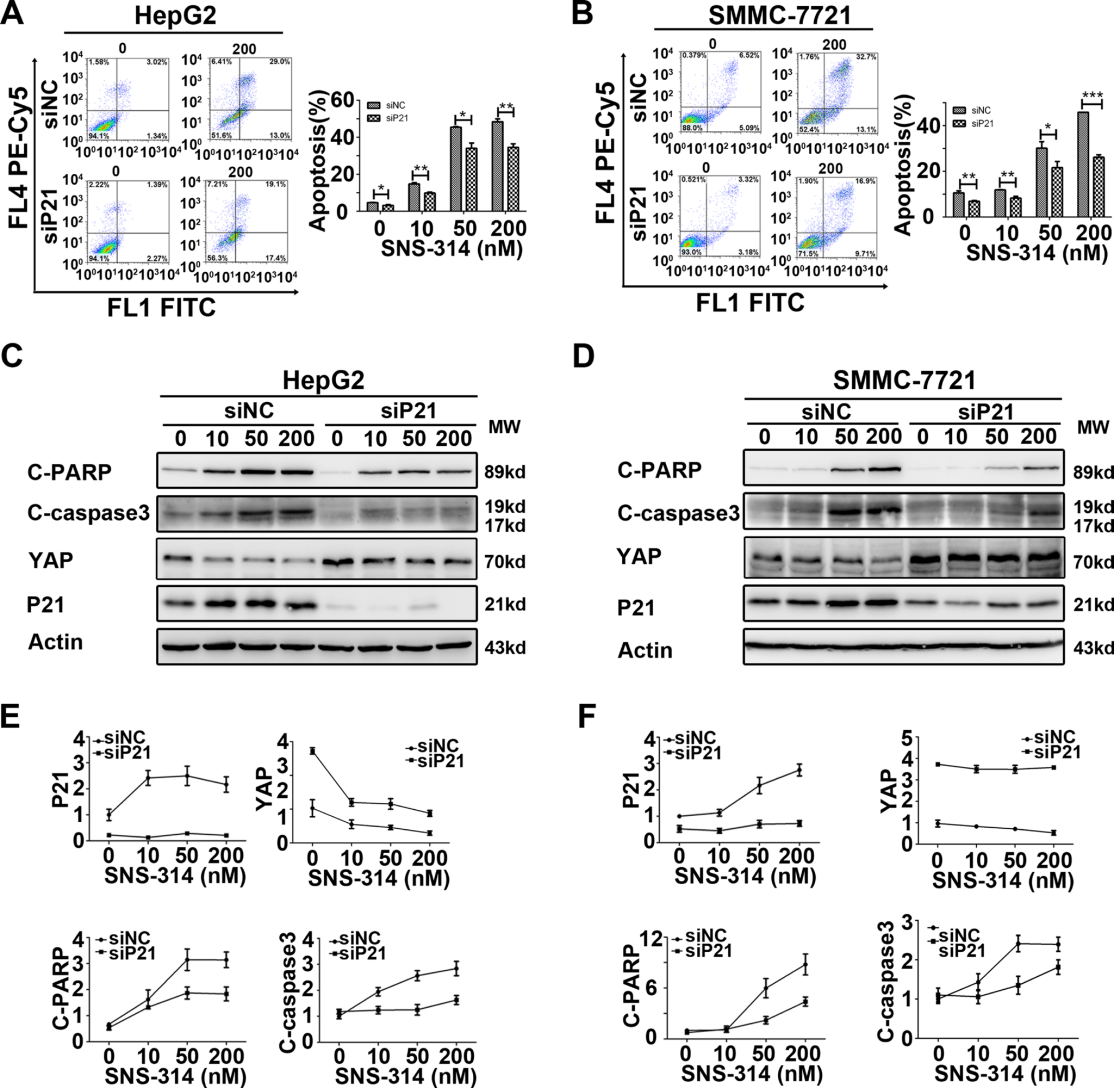
Knockdown of P21 impaired the apoptotic effect of the HCC cell lines treated with SNS-314 (**A–B**) P21 knockdown impaired the apoptosis of HepG2(A) and SMMC-7721(B) cells induced by SNS-314 with indicated concentrations for 96h, suggested by PI and Annexin V staining. (**C–F**) According to WB analysis, knockdown of P21 reduced the activation of cleaved-PARP and cleaved-caspase3, in addition, induced the accumulation of YAP and impaired the reduction of YAP induced by SNS-314 for 96h in HepG2 (C) and SMMC-7721 (D) and the expression of the proteins were quantified by the Image J software in HepG2 (E) and SMMC-7721 (F). FL, Fluorescence; C-PARP, cleaved-PARP; C-caspase3, cleaved- caspase3; Mean ± SD; *p < 0.05, **p < 0.01, ***p< 0.001.

To address the effect of P21 accumulation in SNS-314-induced apoptosis, we did P21 knockdown with siRNA in HepG2 and SMMC-7721 cells, then treated the cells with SNS-314 for 96h. The P21 silencing impaired the cytotoxicity of SNS-314 (data not shown). Similarly, the apoptotic percentage in siP21+SNS-314 treatment group was significantly lower than that of the group transfected with non-sense siRNA control (siNC)+SNS-314 treatment group (Figure 5A–5B). However, there was no statistical difference of the polyploidy (> 4N) formation in the two groups (Supplementary Figure 3). The result suggested P21 accumulation promoted SNS-314-induced apoptosis in HCC.

The impaired activation of c-PARP and c-caspase3 of siP21+SNS-314 treatment was also detected by WB and further confirmed this notion in HepG2 and SMMC-7721 cells (Figure 5C–5D). In addition, P21 knockdown also induced the YAP accumulation and impaired the SNS-314-induced reduction of YAP in HepG2 (Figure 5C) and SMMC-7721 (Figure 5D) cells. Moreover, we further confirmed that siP21 impaired the apoptotic effect in the cells treated with knockdown of Aurora A, or B, or both A and B, respectively (Supplementary Figure 4). These results together indicated the P21 may be invovled in mediating the apoptotic effect of SNS-314 in HCC cells.

## DISCUSSION

**Figure 6:**
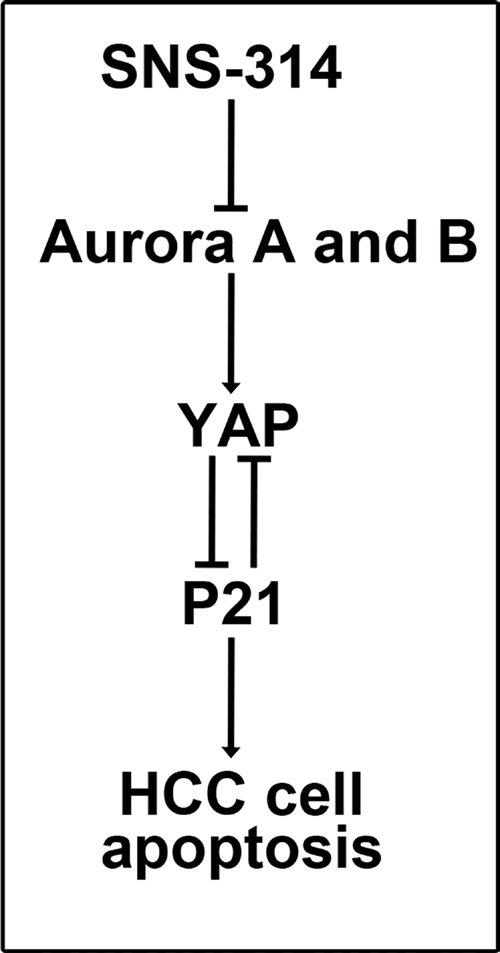
Model of SNS-314 on apoptosis of HCC cells The inhibition of Aurora A and B kinases induced by SNS314 resulted in the reduction of YAP, furthermore, resulted in P21 accmulation which promoted the apoptosis of HCC cells. Also, knockdown of P21 could enhance YAP protein level.

In this study, we first showed Aurora A and B were overexpressed parallelly in HCC, and Aurora A^H^B^H^ was associated with poorer survival. The novel pan-Aurora kinase inhibitor SNS-314 was reported to inhibit both Aurora A and B kinase activities [18], we also showed it suppressed the phosphorylation of histone H3 at Ser10 (data not shown) which was considered as a sensitive and dynamic marker for Aurora inhibition [23]. Here, we found SNS-314 promoted apoptosis of HCC by YAP suppression and induction of P21 accumlation, as shown in Figure 6.

As a member of the serine/threonine protein kinase family, human Aurora kinases (A and B) are essential for proper execution of various mitotic events and important for maintaining genomic integrity [6, 7]. Aurora kinases overexpression are frequently found in various tumors, and correlated with poor prognosis [9, 24–26]. More importantly, our results demonstrated that Aurora A and B overexpressed concomitantly in majority of 24 cases of HCC with a positive linear correlation. And similar result was further confirmed in 141 cases, in addition, the patients with Aurora A^H^B^H^ had the worst survival in this cohort and Aurora A^H^B^H^ was an independent predictor for poor survival. Therefore, we speculated that the anti-tumor effect of pan-Aurora kinases inhibition should be much stronger than that of the specific inhibition for single Aurora kinase. Indeed, our results showed with lower IC50 SNS-314 inhibited HCC growth much strongerly than that of the selective inhibitors, MLN8237 and AZD1152-HQPA.

The Hippo-YAP signaling pathway was shown to be involved in HCC [27], and recent study showed a clear increase of YAP activity in moderately differentiated pediatric HCC [28]. In this study, YAP^H^ was also observed in HCC, particularly that with Aurora A^H^B^H^, and the patients with YAP^H^ and/or Aurora A^H^B^H^ expression had the poorer prognosis, which suggested Aurora kinases and YAP were associated with each other and consistent with previous study which Aurora kinase interacted with hippo pathway [16, 17]. Moreover, we found SNS-314 promoted the polyploidy (DNA>4N) formation and apoptosis, and reduced YAP protein level in HCC. Being associated to poor prognosis and an independent risk factor for HCC survival, YAP reduction induced by SNS-314 could strengthen the inhibitor's anti-tumor effect, which revealed the potential therapy of combined SNS-314 with Hippo-inhibitor in HCC.

Previous study reported that YAP knockdown induced activation of the P53-P21 pathway, thus droved the apoptosis and probably delayed cell cycle progression [21]. As a proliferation inhibitor, the P21 accumulation would block the G1/S and G2/M transitions thus to delay cell cycle progression, in p53-dependent or p53-independent manners, which could protect the cells against apoptosis [29, 30]. Here, our results suggested that SNS-314 reduced YAP level and induced P21 accumulation in HCC, and P21 knockdown impaired SNS-314-induced apoptosis. YAP functioned as a downstream transcriptional co-activator of Hippo pathway [31]; and P21 was also involved in transcriptional regulation by interaction with transcriptional factors [30]. We found P21 silencing induced the accumulation of YAP and weakened the SNS-314-induced reduction of YAP, also YAP silencing induced P21 accumulation, whch indicated that YAP and P21 may interact each other and functioned on transcriptional regualtion. Thus, our findings clearly identified an oncogenic Aurora kinases/YAP/P21 axis in HCC.

In conclusion, Aurora A^H^B^H^ was observed in HCC patients with the poorest survival, and pan-Aurora kinase inhibitor SNS-314 had stronger apoptotic effect on HCC by YAP suppression and induction of P21 accumulation. Our findings not only identified the oncogenic effect of Aurora kinases/YAP/P21 axis in HCC, but also provided solid evidence for SNS-314 as a potential targeted therapy for HCC, and possible combined therapy of SNS-314 plus Hippo pathway inhibitor for HCC.

## MATERIALS AND METHODS

### Culture and regents

The human liver cancer cell lines HepG2, SMMC-7721 and HCC-LM6 were obtained from the Type Culture Collection of Chinese Academy of Sciences (Shanghai, China). HepG2 and HCC-LM6 were cultured in Dulbecco's modified Eagle's medium (Gibco, Life Technologies, Carlsbad, CA, USA) containing 10% fetal bovine serum and 1% penicillin-streptomycin at 37°C with 5% CO_2_. SMMC-7721 was cultured in RPMI-1640 supplemented with 10% fetal bovine serum and 1% penicillin-streptomycin at 37°C with 5% CO_2_. The inhibitors of Aurora kinases, SNS-314, MLN8237 and AZD1152-HQPA, were purchased from Selleck Chemicals (Houston, TX, USA).

### Patient samples

A total of 165 diagnosed HCC samples matched with 163 cases of the corresponding adjacent samples, including 24 pairs of cases for WB analysis and the others for IHC analysis, were collected from the Eastern Hepatobiliary Surgery Hospital, the Second Military Medical University, China, between May 2006 and May 2007. For only the 141 cases of IHC staining, follow-ups were terminated until June 2014. During the follow-up period, a total of 3 patients were lost, which meant 138 patients were eventually available at the final follow-up. Prior written informed consents from all patients and the approval from the Ethics Committees of the Eastern Hepatobiliary Surgery Hospital were obtained.

### Immunohistochemical staining

Using Dako Envisions kit (Dako, Denmark), Immunohistochemical (IHC) staining was performed and detected by Dako Liquid DAB. The slides were heated by microwave (low, medium and high fire, 5 minutes respectively) for antigen retrieval with citrate buffer (pH 6.0), and incubated with primary antibodies (anti-Aurora-A, anti-Aurora-B (Bioworld, USA); anti-YAP (Cell Signaling Technology, USA)) at 37°C for 60 minutes, then incubated overnight at 4°C in moist chambers. The primary antibodies were detected using the Dako EnVision™ Kit. Reaction products were visualized by Dako Liquid DAB. Substrate-Chromagen System and then counterstained with haematoxylin. Negative controls were samples that were incubated with normal goat or mouse serum. The criterion for classifying the staining intensity as follows: Based on four different randomly views with high magnification (magnified 400 times), the percentage of positive cells for nuclear or cytosolic staining were scored as 1 (0 to 25%), 2 (26 to 50%), 3 (51 to 75%), and 4 (76 to 100%). The staining intensity was scored as 1 (negative staining), 2 (weak staining), 3 (moderate staining), and 4 (strong staining). The composite score was obtained by multiplying the percentage grade by the intensity score: + (0~4), ++(5~8), +++(9~12), ++++(13~16). Based on intensity score, + ~ + + + represent low expression and + + + + represents high expression. All results were confirmed using a blind method by at least two pathologists.

### Western blotting analysis

Cell lysates were extracted with the cell lysis buffer (Beyotime, China) and an Enhanced BCA Protein Assay Kit (Beyotime, China) was used to quantified the protein concentration of the cell lysates. Protein samples with 30–50 μg were loaded in each well and separated by 12% PAGE for immunoblotting. And the primary antibodies used were as follows: anti-c-PARP, anti-c-caspase3, anti-P21, anti-Aurora A, anti-Aurora B, anti-YAP, anti-phosphorylated YAP (ser127), anti-phosphorylated YAP (ser397) (Cell Signaling Technology, USA); antiβ-Actin (Kangwei, China).

### RNA interference

Hepatocellular carcinoma cell lines were transfected with siRNA by the Lipofectamine RNAiMAX reagent (Invitrogen, USA) according to the manufacturer's instructions. Briefly, the siRNA and lipofectamine RNAiMAX were incubated separately with opti-MEM (Invitrogen, USA) for 5 min and then mixed together for 20 min at room temperature, then the mixture was applied in the cells plated in dishes and the final concentration of the siRNA is 50 nM.

### ATP cell vialibity assay

Hepatocellular carcinoma cells were plated into the opaque-walled multiwell. According to the manufacturer's instructions, added a volume of CellTiter Reagent (Promega, USA) equal to the volume of cell culture medium present in each well, mixed contents for 2 minutes on an orbital shaker to induce cell lysis, and incubated the plate at room temperature for 10 minutes to stabilize luminescent signal, then recorded the luminescence.

### Hoechst staining

Hepatocellular carcinoma cells were plated into the 6-well plates and treated with SNS-314 for 96 h, then discarded the supernatant, and washed the adherented cells with PBS for twice. Added Hoechst 33342 which was diluted to the concentration of 100 ng/ml with PBS, and incubated for 15 minites at 37°C. After that, the cells were photographed under a fluorescence microscope (Leica, Wetzlar, Germany).

### Cell cycle analysis

Harvested cells and fixed them in 70% ethanol at 20°C overnight, then stained with propidium iodide (36 mg/ml, Sigma) for 30 min, then analyzed by flow cytometry (Beckman Coulter, USA) for cell cycle profile.

### Apoptosis assay

According to the Annexin V-FITC Apoptosis Detection Kit (Invitrogen, USA) instructions, cells were harvested, and washed with PBS, then incubated with Annexin V-FITC and propidium iodide for staining at room temperature for 15 min. Then analyzed by flow cytometry (Beckman Coulter, USA).

### Statistical analysis

The statistical significance of differences between groups was assessed using the GraphPad Prism 5 and SPSS 21.0 softwares. The unpaired two-tailed *t*-test was used for the comparison of parameters between two groups. Categorical data were analyzed by the chi-square or Fisher's exact tests. Correlation between Aurora kinases expression and clinicopathological was performed by the two tailed Mann–Whitney *U*-test or Kruskal–Wallis test. Survival curves were plotted by the Kaplan–Meier method and statistical differences were analyzed using the log-rank test. Cox's proportional hazards regression model was used to analyze independent prognostic factors. For all the tests, three levels of significance (*p < 0.05; **p < 0.01; and ***p < 0.001) were used.

## SUPPLEMENTARY MATERIALS FIGURES


