# Supplementary material for: Targeting high Aurora kinases expression as an innovative therapy for hepatocellular carcinoma
Source: Oncotarget. 2017 Mar 2;8(17):27953–65. doi: 10.18632/oncotarget.15853 (PMC5438621; doi:10.18632/oncotarget.15853)
Supplement: Supplementary file 1 [file oncotarget-08-27953-s001.pdf]

Targeting high Aurora kinases expression as an innovative therapy for hepatocellular carcinoma

Supplementary Materials

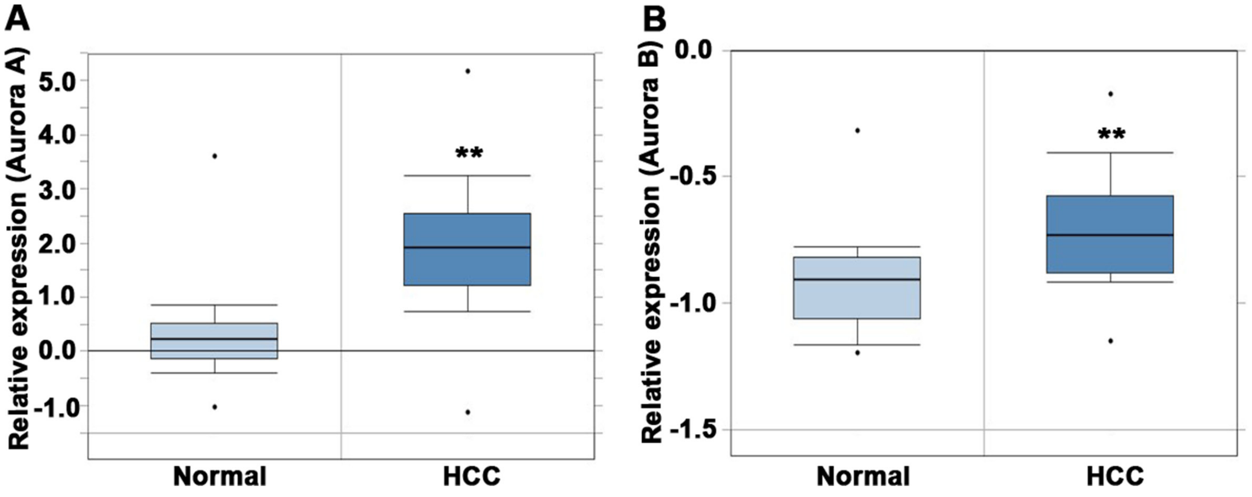

Supplementary Figure 1: The mRNA levels of Aurora A and B in the cancer tissues were higher than that in the adjacent tissues. The mRNA level of Aurora (**A** and **B**) in HCC tissues are obtained from Oncomine Database. The mRNA levels of Aurora A (A) and B (B) in HCC tissues were statistically analyzed with that in the adjacent tissues. A: Normal (n = 220), HCC (n = 225); B: Normal (n = 19), HCC (V38); Mean ± SD; ***p* < 0.01.

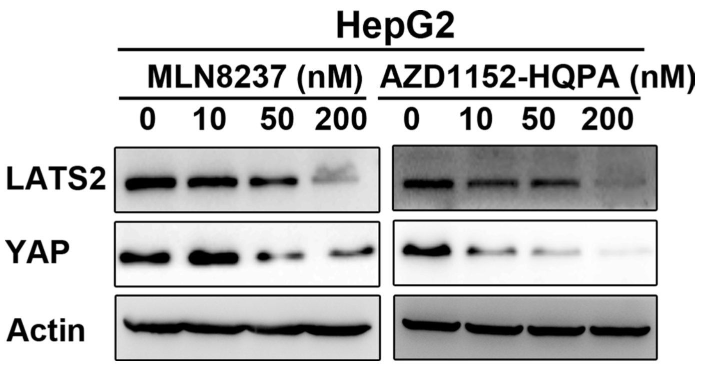

Supplementary Figure 2: The protein level of LATS2 and YAP reduced under the treatment of the selective inhibitors of Aurora kinases. The selective inhibitor MLN8237 and AZD1152-HQPA for kinases Aurora A and B, respectively, reduced the protein level of LATS2 and YAP in a dose-dependent manner in HepG2 cells by Western blot.

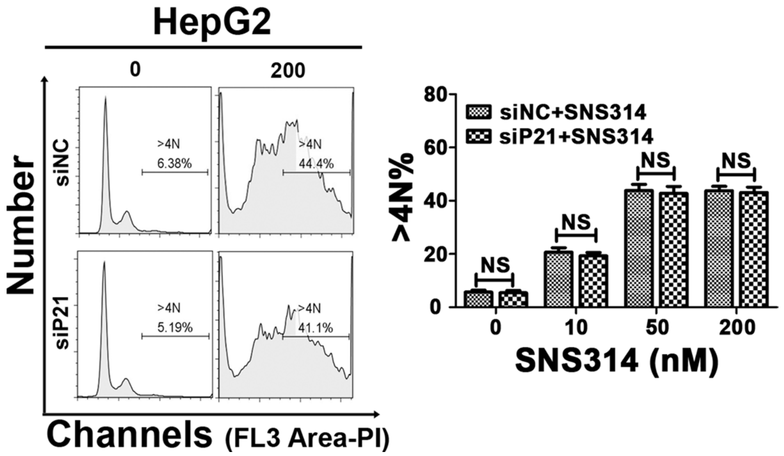

Supplementary Figure 3: Knockdown P21 don't affect the SNS-314-induced polyploidy formation in HCC cells. SNS-314 induced the polyploidy (DNA>4N) in HepG2 cells in a dose-dependent manner, however, no significant difference was detected compared to that in siP21+SNS314 group by the cell cycle analysis with PI staining. FL, Fluorescence; Mean ± SD; NS, no significant.

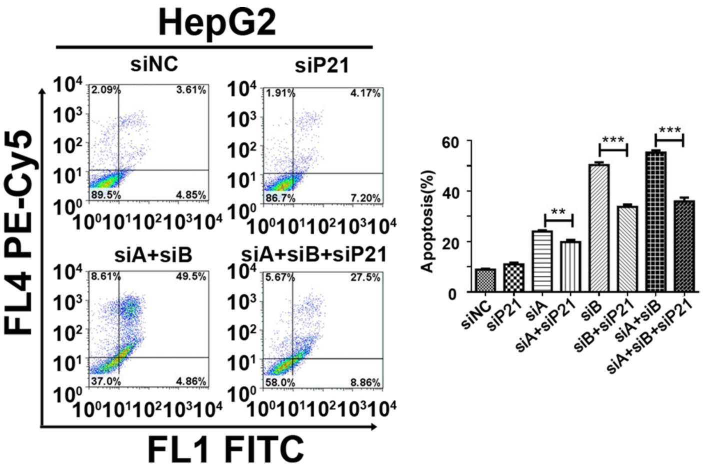

Supplementary Figure 4: siP21 impaired the apoptotic effect of the cells treated with siRNA of Aurora kinases. siP21 impaired the apoptotic effect in the cells treated with siAurora A, or siAurora B, or both of them, respectively, by PI and Annexin V staining and flow cytometry analysis. FL, Fluorescence; Mean ± SD; $**p < 0.01$, $***p < 0.001$.
